# Roles of factor Xa beyond coagulation

**DOI:** 10.1007/s11239-021-02458-8

**Published:** 2021-04-24

**Authors:** Wolfram Ruf

**Affiliations:** 1 Center for Thrombosis and Hemostasis (CTH), Johannes Gutenberg University Medical Center, Langenbeckstr. 1, 55131 Mainz, Germany; 2 Department of Immunology and Microbiology, Scripps Research, La Jolla, CA USA

**Keywords:** Protease activated receptors, Coagulation factor Xa, Tissue factor pathway

## Abstract

Oral anticoagulant therapy has changed by clinical evidence that coagulation factor Xa (FXa) can be safely and effectively targeted for thromboprophylaxis. Because thrombotic and thrombo-inflammatory diseases are frequently caused by excessive activation of the tissue factor (TF) pathway, activation of FX by the TF-FVIIa complex is of central importance for understanding the roles of FXa in thrombosis and hemostasis and functions beyond blood coagulation. Recent data showed that the nascent product FXa associated with TF-FVIIa not only supports hemostatic cofactor VIII activation, but also broadly influences immune reactions in inflammation, cancer, and autoimmunity. These signaling functions of FXa are mediated through protease activated receptor (PAR) cleavage and PAR2 activation occurs in extravascular environments specifically by macrophage synthesized FX. Cell autonomous FXa-PAR2 signaling is a mechanism for tumor-promoting macrophage polarization in the tumor microenvironment and tissue penetrance of oral FXa inhibitors favors the reprogramming of tumor-associated macrophages for non-coagulant therapeutic benefit. It is necessary to decipher the distinct functions of coagulation factors synthesized by the liver for circulation in blood versus those synthesized by extrahepatic immune cells for activity in tissue milieus. This research will lead to a better understanding of the broader roles of FXa as a central regulator of immune and hematopoietic systems.

## Highlights

- FXa has distinct functions as nascent product of TF-FVIIa and in the FVa-FXa prothrombinase complex.
- Release of FXa from TF-FVIIa is linked to complement- and protein disulfide isomerase-dependent allosteric regulation of TF.
- FXa assembled with TF-FVIIa activates FVIII and thereby hemostatic coagulation as well as PAR2 signaling in immunity.
- Regulated synthesis of FX by innate immune cells controls macrophage function in extravascular milieus.

This brief review in conjunction with the symposium ‘Factor X: From Thrombokinase to Oral Anti-coagulants and Beyond’ will summarize the role of coagulation factor (F) X in the extrinsic coagulation pathway. Extrinsic coagulation activation by the cell surface receptor tissue factor (TF), originally described as tissue thromboplastin, is tightly linked to cellular effects that range from regulation of cell motility to cell activation and quiescence specifically in the contexts of immunity and regeneration. Our current understanding of the basic biochemistry and interactions of FXa that allow for these contributions to cellular processes will be reviewed here.

## FX is the preferred substrate for the TF initiation complex

The cloning of the coding sequence for the coagulation initiator TF, which serves as the cell surface receptor and allosteric cofactor for FVIIa [1], initially indicated a structure distinct from the plasma coagulation cofactors V and VIII that support FXa and FIXa, respectively. Structure prediction algorithms subsequently suggested an evolutionary origin of TF as a member of the cytokine receptor family. Mutational and structural studies rapidly confirmed the fold of TF into two fibronectin type III-like domains typical for these immune receptors and elucidated the structure–function relationships of the interaction between TF and FVIIa [2]. Mutagenesis of TF initially identified an exosite in the carboxyl-terminal extracellular domain of TF. This exosite was not involved in binding of FVIIa and was critically important for the activation of FX to FXa, but much less so for the activation of FIX [3]. Modeling of macromolecular FX docking to the TF-FVIIa coagulation initiation complex provided a view of the extended contacts between the three proteins and the predicted interactions of FX were consistent with extensive prior and model-probing mutagenesis [2].

**Fig. 1:**
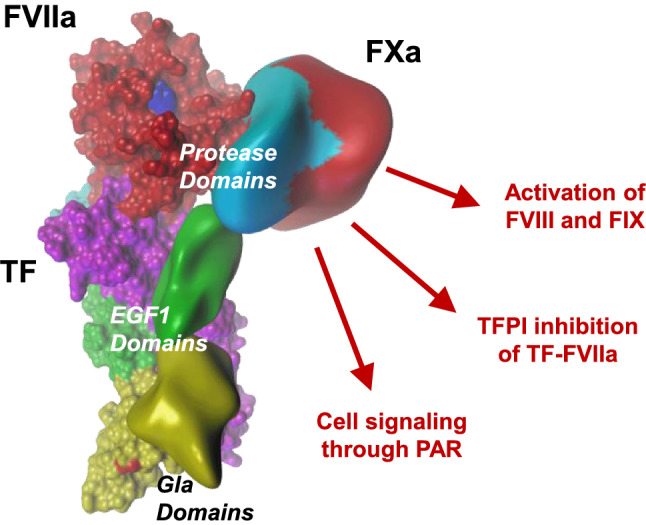
Assembly of FXa with the TF-FVIIa complex and functions of nascent product related to coagulation and cell signaling. FX interacts with an extended interface created by exosites on TF and FVIIa, but does not dock into the activate site of FVIIa (shown in blue). This stable complex assembly allows for cleavage of the activation peptide bond in zymogen FX, but also for thethering of the nascent product FXa which is involved in regulation of TF function by TFPI, the activation of FVIII in the antihemophilic pathway and cell signaling through PARs. Details of the assembly interaction and active sites can be found in reference [2]

Macromolecular substrate FX interacts with TF-FVIIa primarily through multiple and distinct exosites remote from the catalytic cleft of FVIIa (Fig. 1). Specifically, the γ-carboxyglutamic acid-rich (Gla) domains of substrate FX and enzyme FVIIa make contacts with each other as well as the carboxyl-terminal domain of TF. The epidermal growth factor (EGF) 1 domains of FX and FVIIa bind through energetically significant contacts to TF but make little contact with each other. Whereas the protease domain of FVIIa interacts with TF to allow for the allosteric activation of protease function [1], the protease domain of FX and the tightly associated EGF-2 domain primarily interact with an allosterically regulated exosite region of the protease domain of FVIIa (Fig. 1). These extended interactions allow for alignment of macromolecular substrate with the active site of FVIIa, into which the flexible activation peptide of FX docks for cleavage. However, the interactive surface of the FX protease domain with FVIIa is largely unaffected by zymogen to enzyme conversion of the nascent product FXa [2]. Thus, FXa can remain associated with TF-FVIIa before being released for the subsequent reactions in the common coagulation pathway.

**Fig. 2:**
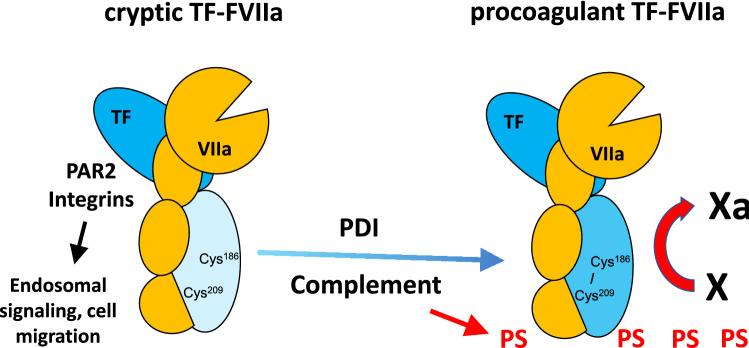
Regulation of TF-FVIIa complex functions by complement and PDI. The allosteric Cys^186^-Cys^209^ control trafficking of TF-FVIIa and FX turnover. In the cryptic state, TF-FVIIa signals through PAR2 in the endosome and regulates cell migration by associating with integrins. Complement activation induces thiol-disulfide exchange involving PDI and in the context of phosphatidylserine (PS) exposure leads to allosteric activation of TF favoring FX turnover and coagulation

Despite the detailed elucidation of the structural basis for TF-FVIIa recognition of FX, subsequent studies have shown that cellular and posttranslational mechanisms are crucial determinants for the distinct functions of the TF initiation complex in thrombosis, hemostasis and cell signaling. Monocytes and macrophages express TF on the surface in an inactive or cryptic form that is converted to fully procoagulant TF in the context of inflammation (Fig. 2). This process requires thiol-disulfide exchange and involves protein disulfide isomerase (PDI) that modify the conformation of the TF extracellular domain through chaperone activity of PDI and reshuffling of the allosteric disulfide bond exposed in the carboxyl-terminal domain of TF [4]. The TF Cys^186^-Cys^209^ disulfide can be S-nitrosylated, reduced or converted to mixed disulfides of TF Cys^209^ with thioredoxin (Trx) or glutathione [4]. In the presence of PDI, affinity of TF for FVIIa is high and turnover of macromolecular substrate FX is favored [5].

Complement activation is a crucial trigger for thiol-disulfide exchange pathways and involves antibody binding to monocyte receptors and activation of complement factor 3 in the antiphospholipid syndrome [6, 7] or antibody independent pathways in venous thrombosis [8]. Importantly, conversion of TF to a prothrombotic state requires the cell surface translocation of phosphatidylserine (Fig. 2), although the exposure of negatively charged phospholipids in the absence of PDI is not sufficient for TF prothrombotic activity on extracellular vesicles [5]. Phosphatidylserine exposure can be induced by several inflammatory mechanisms, including complement C5b-C7 membrane insertion leading to thrombosis [4], conventional [5] or non-conventional [9] inflammasome activation in sepsis, or thrombin-PAR1/PAR2 signaling leading to acid sphingomyelinase activation for prothrombotic TF generation and inflammation in the antiphospholipid syndrome [6, 7].

## Function of FXa assembled with the TF-FVIIa complex

The prothrombotic and hemostatic functions of FXa after release from the TF-FVIIa complex and assembly with the plasma coagulation cofactor Va are well characterized, but FXa fulfills distinct roles while associated as a nascent product with TF-FVIIa. FXa can elicit such immediate proteolytic roles because the active site of FXa is not in direct contact with TF-FVIIa and therefore is available to recognize substrates and inhibitors (Fig. 1). The interaction of the second Kunitz-type protease inhibitory domain of TF pathway inhibitor (TFPI) with the active site of FXa is a crucial step required for efficient inhibition of the TF-FVIIa complex by TFPI Kunitz domain 1. This inhibitory complex formation is essential for controlling the activity of TF in coagulation as well as cell signaling [10]. Although imaging has shown that the TF-FVIIa-FXa-TFPI complex forms and persists on monocyte cell surfaces [7], biochemical evidence indicates that TFPI inhibition is not instantaneous and fails to block all functions of nascent product FXa [11].

**Fig. 3:**
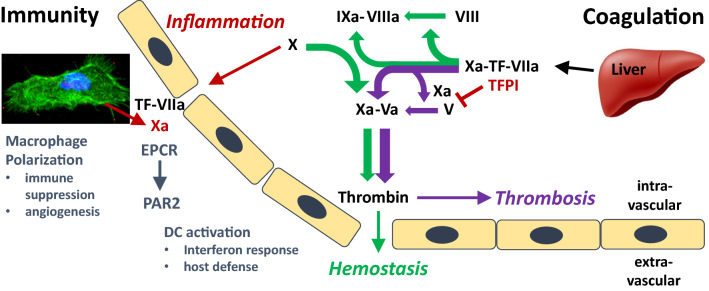
FXa functions in TF-FVIIa initiated intravascular coagulation, hemostasis, and extravascular immune regulation. TF-FVIIa activated nascent product Xa initiates the intrinsic pathway by cleaving FVIII (green arrows), whereas FXa released from TF-FVIIa activates FV to directly form the prothrombinase in the common coagulation pathway (purple arrows). TFPI regulates FXa-dependent FV activation as well as controls TF-FVIIa in a FXa-dependent manner. FX activated on dendritic cells (DC) induces host defense-related interferon responses through PAR2 signaling in the context of toll-like receptor 4 activation and dependent on the endothelial protein C receptor (EPCR). FX can also by synthesized by macrophages and extravascular cell-autonomous FXa-PAR2 signaling regulates macrophage polarization involved in tumor immune evasion in tissue milieus

This conclusion is supported by studies with a protease domain mutant of FVIIa (FVIIa E154A). This mutant can convert FX to FXa but is highly defective in releasing the nascent product FXa. Thrombin generation in plasma initiated by this mutant requires the anti-hemophilic FIX and FVIII. Importantly, the nascent FXa generated by the mutant FVIIa complex with TF directly activated FVIII, but not FV [11]. Remarkably, even supraphysiological concentrations of TFPI could not block nascent product FXa mediated FVIII activation, although TF-FVIIa generated FXa was prevented from assembling into the prothrombinase complex (Fig. 3). Consistent with these results, studies in reconstituted blood showed that the mutant was almost as effective as wild-type FVIIa to initiate FVIII-dependent platelet and fibrin deposition under flow. Thus, nascent product FXa may have a distinct role in triggering the FVIII-FIX pathway involved in hemostasis in contrast to the functions of FXa in the prothrombinase and thrombosis.

Nascent FXa is also critically involved in cell signaling processes. As discussed above, high affinity for FVIIa characterizes TF which efficiently activates FX. Whereas the TF-FVIIa complex at high concentrations of FVIIa can directly activate PAR2 and supports integrin-dependent endosomal signaling [7, 12] (Fig. 2), low concentrations of FVIIa elicit cell signaling only when FX is present [13]. Signaling of the nascent product FXa is therefore integrated in cell surface TF-initiated coagulation. TF-FVIIa-Xa ternary complex signaling in endothelial, smooth muscle and cancer cells also requires the endothelial cell protein C receptor (EPCR) (Fig. 3). EPCR binds the Gla-dominas of protein C (PC), FVII and FX, but TF-FVIIa-FXa signaling did not require Gla-domain interactions of FVIIa with EPCR [14]. Thus, FXa can recruit additional receptors, in this case EPCR, to modulate the signaling functions of TF. Remarkably, although EPCR mediates endothelial cell protection in inflammation through PAR1 signaling, EPCR recruited to the TF-FVIIa-FXa complex alters the lipopolysaccharide (LPS) response of dendritic cells through PAR2 signaling [15]. In the context of toll like receptor signaling, nascent product FXa engaging TF-FVIIa as well as EPCR is crucial for the expression of type I interferon regulated genes that participate in diverse host defense and autoimmune signaling pathways [6].

The innate immune signaling role of nascent FXa in the TF coagulation initiation complex is surprisingly regulated by the anticoagulant PC pathway. The thrombin-thrombomodulin complex activates PC in an EPCR-dependent manner on endothelial cells. Activated PC typically suppresses coagulation by inactivating the plasma coagulation cofactor VIIIa dependent on non-activated FV and anticoagulant protein S. This pathway is known to be defective in FV_Leiden_ patients which present with the clinical diagnosis of PC resistance. The complex of FV, protein S, and activated PC also suppresses TF-FXa-PAR2 signaling in the context of the endotoxin-induced upregulation of interferon-regulated genes. Because FXa engages EPCR in this signaling complex, the anticoagulant pathway presumably exerts the regulatory function by competition of activated PC with FXa ligand occupancy of EPCR [16]. These experiments emphasize that pro- and anticoagulant pathways are integrated into innate immune responses to microbial or endogenous danger signals.

## Extrahepatic synthesis of FXa as a regulator of the immune system

PARs are sensors for proteolytic activity and PAR activation by coagulation proteases serves as an injury signal for immune cells circulating in blood or lymphatics. Several lines of evidence point to important functions of coagulation proteases activating PARs in extravascular locations (Fig. 3). Although vascular leakage in the context of inflammation may allow coagulation proteases to exit the blood stream, cells in tissue micromilieus can synthesize certain coagulation proteases for regulatory functions through PARs. In the bone marrow niche, FXa can be derived from megakaryocytes and form the prothrombinase to generate extravascular thrombin. The balance between thrombin and the anticoagulant activated PC controls hematopoietic stem and progenitor cell (HSPC) mobilization and quiescence through PAR1 signaling [17]. Being at the apex of the hematopoietic lineages, HSPC differentiation and responses to infection and injury are thereby directly controlled by the coagulation cascade.

In addition to the extrinsic coagulation cofactor and receptor TF, the plasmatic factor V is also synthesized by macrophages, enabling rapid thrombin generation in the peritoneal cavity following infection and in other tissue milieus during thrombo-inflammatory processes [18]. Tissue resident macrophages and tumor-associated macrophages (TAM) also synthesize FVII and FX [19], indicating that initiation of coagulation by TF can lead to the formation of a functional FVa-FXa prothrombinase solely by cell autonomous coagulation factor synthesis. Because TAM support angiogenesis, metastasis and immune evasion, it became a pertinent question whether cell autonomous synthesis of coagulation factors alters macrophage phenotypes in cancer and potentially other diseases.

Studies were conducted in immune competent mice with syngeneic tumor models which allowed for the analysis of adaptive immunity and evaluation of cytotoxic tumor cell killing. Prior clinical trials in cancer patients showed only marginal therapeutic benefits of low molecular weight heparin (LMWH) on tumor growth and cancer progression. We compared the efficacy of LMWH with the FXa inhibitor rivaroxaban in various tumor models [19] and found that both anticoagulants were highly efficacious in preventing spontaneous metastasis, a process that requires intravascular thrombin and platelet activation. However, only rivaroxaban, but not LMWH suppressed tumor growth, indicating that the tissue penetrance of small oral FXa inhibitors targeted a crucial extravascular function of FXa. Indeed, we detected the expression of FX along with FVII, but not other coagulation factors in monocytes and macrophages within the tumor microenvironment [19, 20].

Because deletion of PAR2 in monocytes and macrophages impaired tumor growth, we first evaluated the role of FXa signaling by generating a PAR2 mutant mouse model in which FXa can no longer cleave PAR2. In these mice, various syngeneic tumor models grew slower and tumor eradicating cytotoxic lymphocytes were enriched in the tumor stroma [19]. In addition, genetic deletion of FX from macrophages produced a similar reduction in tumor growth along with improved anti-tumor immune responses, but rivaroxaban treatment had no additional effect in mice with macrophage FX deficiency. Thus, in immune competent mice we uncovered novel immune suppressive roles for FXa activating PAR2 that were not seen in previous studies with immune deficient mice.

In addition, when isolated macrophages were stimulated with tumor cell supernatant, we found that the resulting immune-evasive macrophage polarization was prevented by macrophage deletion of FX, rendering PAR2 insensitive to FXa cleavage, or by addition of the direct FXa inhibitor rivaroxaban. We also saw a similar reprogramming of macrophages in the tumor stroma under these experimental condition, demonstrating that cell autonomous FXa-PAR2 signaling determines TAM phenotypes and that this signaling pathway can be therapeutically targeted by tissue-penetrating oral FXa inhibitors [19].

## Concluding remarks

The definition of FXa as the central enzyme in the common plasmatic coagulation pathway by the work of Haskell Milstone has since then evolved to an understanding of FXa function in cellular signaling processes that crucially regulate immunity. Serine proteases are essential components in the activation of the innate immune defense toll pathway in drosophila. FXa generated and associated with TF-FVIIa appears to perpetuate this central function of antimicrobial host defense by cleaving PAR2 in vertebrates. The regulation of FX activation and its shutdown by inhibitory pathways thus centrally affect many cellular functions beyond the clotting reactions studied extensively in the last century.
